# Orthobunyaviruses in the Caribbean: Melao and Oropouche virus infections in school children in Haiti in 2014

**DOI:** 10.1371/journal.pntd.0009494

**Published:** 2021-06-16

**Authors:** Maha A. Elbadry, Ricardo Durães-Carvalho, Gabriela M. Blohm, Caroline J. Stephenson, Julia C. Loeb, Sarah K. White, Taina Telisma, Sonese Chavannes, Valery M. Beau De Rochars, Marco Salemi, J. Glenn Morris, John A. Lednicky

**Affiliations:** 1 Department of Environmental and Global Health, College of Public Health and Health Professions, University of Florida, Gainesville, Florida, United States of America; 2 Emerging Pathogens Institute, University of Florida, Gainesville, Florida, United States of America; 3 Laboratory of Virology, University of Campinas (UNICAMP), Campinas-SP, Brazil; 4 Christianville Foundation, Gressier, Haiti; 5 Department of Health Service Research, Management and Policy, College of Public Health and Health Professions, University of Florida, Gainesville, Florida, United States of America; 6 Department of Pathology, College of Medicine, University of Florida, Gainesville, Florida, United States of America; 7 Department of Medicine, College of Medicine, University of Florida, Gainesville, Florida, United States of America; Aix-Marseille Universite, FRANCE

## Abstract

We report the identification of two orthobunyaviruses, Melao virus (MELV) and Oropouche virus (OROV), in plasma specimens from Haitian children with acute febrile illness who presented during outbreaks caused by alpha- and flaviviruses in 2014. Heretofore not described as a human pathogen, MELV was isolated in cell culture from the plasma of five case patients. OROV RNA was detected in the plasma of an additional child, using an unbiased sequencing approach, with phylogenetic inference suggesting a close relationship with strains from Brazil. Abdominal pain was reported by four case patients with MELV infections, with lymphadenopathy noted in two cases. Our findings document the occurrence of these orthobunyaviruses within the Caribbean region and highlight the critical importance of surveillance with viral genome sequence analyses to identify outbreaks caused by these and other emerging viruses.

## Author summary

Melao and Oropuche virus infections were detected in Haitian children who developed acute febrile illnesses in year 2014. As these viruses were not previously known to circulate in Haiti, our findings highlight the critical importance of surveillance to identify outbreaks caused by these and other emerging viruses.

## Introduction

The International Committee on the Taxonomy of Viruses currently lists >180 viruses in the genus *Orthobunyavirus* (order *Bunyavirales*, family *Peribunyaviridae*) [1,2]. While little data is available for many of these viruses, several are well known causes of human disease, including La Crosse virus (LACV) and Jamestown Canyon virus (JCV), which are currently the second and third most common causes, respectively, of arbovirus neuroinvasive disease in the United States [3,4]. Others are known to cause fever and rash syndromes and based on serologic studies of the general population in endemic regions, it is likely that asymptomatic infection is common [5]. Orthobunyaviruses are enveloped and possess a genome composed of three segments (small, medium, and large) of negative-sense single-stranded RNAs. The small segment encodes the nucleoprotein (N) and depending on the virus, may also encode a non-structural protein (NSs), the medium segment encodes two glycoproteins and a non-structural protein (NSm), and the large segment encodes the RNA-dependent RNA polymerase [6,7].

Melao virus (MELV) was first isolated from *Aedes* and *Psorophora spp*. mosquitoes captured in the Melajo forest in Trinidad [8,9]. Neutralizing antibodies against MELV or a highly related virus have been identified in horses and humans, suggesting that MELV can cause infections leading to antibody production in these hosts [10,11]. However, there has been no prior documentation of MELV-associated human illness. In contrast, Oropouche virus (OROV) causes Oropouche febrile illness, which is presently one of the common arbovirus-associated febrile illnesses in Brazil. This virus is known to cause meningoencephalitis [12]. First isolated in Trinidad, there have now been more than 30 epidemics and over half a million clinical cases attributed to OROV in Brazil, Peru, Ecuador, and Panama [12].

We report isolating MELV from, and finding OROV RNA in, plasma samples collected from children with acute febrile illness in a school cohort in Haiti, with virus identities confirmed by genome sequence analyses. To our knowledge, neither virus has been previously reported in Haiti, and this is the first time we have detected orthobunyaviruses in febrile patients at our Haitian study sites.

## Materials and methods

### Ethics statement

The studies of this report were approved by the Institutional Review Board (IRB) at the University of Florida (IRB201300609) and the Haitian National IRB. Only children presenting with acute febrile illness, defined as a history of fever and/or a measured temperature > over 37.5°C in the clinic with no localizing symptoms or signs (i.e., no respiratory, skin, or urinary signs or symptoms) were offered an opportunity to enroll in the study. Written informed consent for study participation was obtained from parents, with assent from children. After enrollment, clinic health care providers recorded clinical data in a study questionnaire, and a sample of venous blood was collected for microbiology analyses from the children.

### Study population

This study centered on a cohort of approximately 1,250 school children that our University of Florida (UF) research group monitored in the Gressier region of Haiti through 2019. The children attended one of four schools operated by the Christianville Foundation, and had free access to medical care through a school-based clinic. Data on the identification of alpha- and flavivirus infections among children within this school cohort have been previously reported [13–15,21,24].

### Plasma samples

Students provided a 3 mL sample of venous blood that was drawn into 13 x 100 mm acid-citrate-dextrose solution B blood collection tubes (Becton, Dickinson, and Company, Franklin Lakes, NJ, USA). The blood samples were promptly transported to the UF laboratory in Gressier, Haiti, where they were centrifuged to pellet blood cells, and plasma retrieved and aliquots thereof transferred to cryostorage tubes and stored at -80°C pending microbiology analyses.

### RT-PCR screens

In accordance with our arbovirus surveillance work in Haiti, RNA was extracted from virions in plasma using a QIAamp Viral RNA Mini Kit (Qiagen Inc., Valencia, CA) and screened by RT-PCR for chikungunya virus (CHIKV), dengue virus (DENV), and zika virus (ZIKV) genomic RNAs (vRNAs). The RT-PCR tests were redundantly performed, first by using the Zika-Chikungunya-Dengue (ZCD) multiplex assay of Waggoner *et al*. [16] as an initial screen, then for added sensitivity of detection, by using virus-specific tests for CHIKV [17], DENV-1 to -4 [18], and ZIKV [19]. Samples that tested negative for CHIKV, DENV-1 to -4, and ZIKV, were further analyzed for other viruses.

### Malaria parasites

An aliquot of each plasma sample was screened for malaria parasites using the CareStart HRP2 rapid diagnostic test (RDT). This CareStart RDT has a high sensitivity and moderate specificity when compared with blood smear microscopy for diagnosis of malaria [20].

### Virus isolation in cell cultures

Following arbovirus isolation methods we have employed in the past [13–15,21], 25 plasma samples that were negative for CHIKV, DENV1-4, and ZIKV by RT-PCR and for malaria parasites were further screened by virus isolation attempts in two cell lines that are permissive and susceptible to many arboviruses that affect humans. Virus isolation work was initiated in a BSL3 laboratory as a precautionary measure in case an agent requiring work in such a facility was isolated. Briefly, LLC-MK2 (CCL-7) and Vero E6 (CRL-1586) that had been obtained from the American Type Culture Collection (ATCC, Manassas, VA, USA) and demonstrated to be free of mycoplasma and adventitious viruses [22] were propagated as monolayers in T25 cell culture flasks at 37°C and 5% CO_2_ in advanced Dulbecco’s Modified Eagle’s Medium (aDMEM, ThermoFisher Scientific) supplemented with 2 mM L-Alanyl-L-Glutamine (GlutaMAX, ThermoFisher Scientific), antibiotics (PSN; 50 μg/mL penicillin, 50 μg/mL streptomycin, 100 μg/mL neomycin) (ThermoFisher Scientific) and 10% gamma-irradiated low-IgG fetal bovine serum (HyClone, Logan, UT) until they reached 80% confluency. Next, aliquots of thawed plasma specimens were filtered using sterile 0.45 μm pore-diameter nylon membrane filters (Acrodsic syringe-filters, Pall Corporation), and 50 μL of filtered plasma used to inoculate LLC-MK2 and Vero E6 cell cultures in just enough complete aDMEM to cover the cells [13]. After one hour at 37°C and 5% CO_2_, four ml of media was added to the inoculated cells. The following day, the cell growth medium was removed and replaced with maintenance medium consisting of aDMEM containing GlutaMAX, PSN, and 3% FBS. Mock-inoculated cells were maintained in parallel. Thereafter, the LLC-K2 cells were refed every 3 days and the Vero E6 cells every 5 days. Seven days post-inoculation (dpi), the cells in T25 flasks were split into T75 flasks, where they were maintained on the same re-feeding schedule using maintenance medium. At 18 dpi, the cells in each T75 flask were split 1:3 into three T75 flasks, and once again maintained on the same re-feeding schedule. The cells were observed for development of virus-induced cytopathic effects (CPE) for 30 dpi at 37°C before being considered negative for virus isolation. In the event that the cells were persistently infected without showing signs of CPE, as sometimes observed with DENV and ZIKV, RT-PCR screens with the ZCD assay were performed on RNA extracted from cell culture media 15 and 30 dpi. Cells that showed signs of CPE were scraped when at least 50% of the cells had died, and the scraped cells, cell lysate, and spent cell culture media transferred (together) into cryovials and archived at -80°C pending further analyses.

### RT-PCR screens for S. American alpha- and flaviviruses other than CHIKV, DENV, and ZIKV

As RT-PCR tests indicated that the agent that caused CPE in LLC-MK2 and Vero E6 cell was not CHIKV, DENV-1 to -4, or ZIKV, vRNA from the cell culture media was screened with a duplex RT-PCR for other alphaviruses (Venezuelan equine encephalitis -, Eastern equine encephalitis -, Western equine encephalitis -, Aura- and Mayaro viruses) and flaviviruses (Yellow fever -, Saint Louis encephalitis -, Bussaquara -, Ilheus -, and Rocio viruses) using the nested RT-PCR assay of de Morais Bronzoni et al. [23,24].

### Unbiased nucleotide sequence amplification and detection of MELV RNA purified from virions in cell culture media

An aliquot (300 μl) of spent Vero E6 culture media from the first isolate, designated Haiti-1/2014 (Table 1) was treated with cyanse nuclease (RiboSolutions, Inc., Cedar Creek, TX) to degrade nucleic acids external to that packaged (and thus protected) in virions [13]. vRNA (and viral DNA) was subsequently extracted from the cyanase-treated virions in the plasma using a QIAamp Viral RNA Mini Kit (Qiagen Inc.) and the purified RNA/DNA preparation subjected to rRNA depletion using a QIAamp Viral RNA Mini Kit (Qiagen Inc.). Since it was not known if there was a DNA or RNA virus, both PCR alone and RT-PCR amplifications were performed. For RT-PCR, first-strand synthesis was performed using random hexamers and an Accuscript High Fidelity 1st strand cDNA kit (Agilent Technologies, Santa Clara, CA), and PCR performed using random hexamers and One Taq DNA polymerase (New England Biolabs, Ipswich, MA, USA). These procedures resulted in the formation of amplicons as evidenced by the presence of discreet bands viewed using after electrophoresis in a 2% agarose gel stained with ethidium-bromide (EtBr). The PCR amplicons were purified from the PCR reactions using a Qiagen QIAquick PCR purification kit, then A-tailed with Taq DNA polymerase (New England Biolabs) as a precaution since One Taq is a mixture of a high-fidelity polymerase and Taq, and therefore not all amplicons may contain 3’ A-tails. The A-tailed PCR amplicons were then TA-cloned using a commercial kit (pCR2.1 vector with one shot TOP10 chemically competent *E*. *coli* cells, ThermoFisher Scientific K204001), and the inserts in 40 clones (20 clones from PCR, 20 clones from RT-PCR) Sanger sequenced.

**Table 1:** GenBank accession nos. and complete or partially complete genome segment lengths (rnt) of MELV/Homo sapiens/Haiti/2014 isolates 1–4.

MELV isolate	Genome segment	GenBank No.	Genome length^a^	% Nt identity with MELV TRVL^b^
Haiti-1/2014	L	MN264270	6,975	6932/6957(99%)
	M	MN264271	4,498	4489/4498(99%)
	S	MN264272	1,099	964/969(99%)
Haiti-2/2014	L	MN268722	6,952	6927/6952(99%)
	M	MN268723	4,498	4489/4498(99%)
	S	MN268724	964	964/969(99%)
Haiti-3/2014	L	MN268725	6,918	6891/6918(99%)
	M	MN268726	4,493	4483/4492(99%)
	S	MN268727	1,072	964/969(99%)
Haiti- 4/2014	L	MN268728	6,942	6917/6942(99%)
	M	MN268729	4,488	4479/4488(99%)
	S	MN268730	1,072	964/969(99%)

^a^Complete or partial genome sequence length (ribonucleotides) obtained by sequencing.

^b^Percent nucleotide identity with Melao virus reference strain TRVL: L gene, NC_043634.1; M gene, NC_043633.1; S gene, NC_043635.1.

### Sequencing of MELV vRNAs

After determining the vRNA from the first isolate that formed unusual CPE was from MELV, an RT-PCR test to detect the 249 bp MELV sequence described above was used to screen the other four isolates, since they formed similar CPE, and they were positive for the insert and thus also presumably isolates of MELV. The isolates were therefore deemed safe to work with in a BSL2 facility and transferred to our BSL2 laboratory. Therein, two approaches were used to attempt to determine the genome sequences of the virus isolates: (a) Sanger sequencing, and (b) next-generation sequencing (NGS). A gene walking approach and 5/3 RACE (rapid amplification of cDNA ends) were used to Sanger sequence the virus. Briefly, targeted overlapping (tiled) sequences (< 800 bp amplicons) were amplified using Accuscript High Fidelity reverse transcriptase in the presence of SUPERase-In RNase inhibitor (Ambion, Austin, TX), followed by PCR with Q5 high-fidelity DNA polymerase (New England Biolabs) with denaturation steps performed at 98°C. The primers that were optimized for the gene walking sequencing approach are listed in S1 Table; a red letter in M segment primer 1 Fm denotes a nucleotide difference from the corresponding nt position in MELV M segment reference sequence NC_043633.1. To obtain the 5′ and 3′ ends of the three MELV genomes, a 5′ and 3′ system for RACE was used per the manufacturer’s protocols (Life Technologies, Carlsbad, CA, USA); for these reactions, the 3’ vRNA ends were A-tailed using a Poly(A)-Polymerase (ThermoFisher Scientific, USA) prior to 3’ RACE. Noteworthy, RACE was not successful for all samples. All PCR amplicons were purified, sequenced bi-directionally using Sanger Sequencing, and the sequences assembled with the aid of Sequencher DNA sequence analysis software v2.1 (Gene Codes, Ann Arbor, MI, USA).

### Quantification of infectious MELV load in plasma

A 50% endpoint dilution assay (TCID_50_ assay) [25] in Vero E6 cells grown in 96-well plates was used to quantify infectious MELV loads in plasma. Briefly, newly confluent cells Vero E6 cells in aDMEM, supplemented with 2mM GlutaMAX), PSN, and 10% gamma-irradiated low-IgG fetal bovine serum were inoculated with ten-fold dilutions of plasma. After overnight-incubation, the cell growth medium was removed and replaced with maintenance medium consisting of aDMEM containing GlutaMAX, PSN, and 3% FBS. The cells were thereafter refed with maintenance medium every 5 days and observed over 16 dpi. Mock-inoculated cells were maintained in parallel, and virus isolated from this work was used as a positive control. To verify that MELV had been isolated, vRNA was isolated from one of the wells at the highest dilution causing CPE and tested by RT-PCR using primer pair 3Fm and 3Rm, as listed in S1 Table, M segment.

### Unbiased sequence amplification and detection of OROV RNA purified from plasma

Aliquots (300 μl) of 20 out of the 25 plasma samples for which virus was not isolated in cell cultures were treated with cyanse nuclease as mentioned above, and nucleic acids subsequently extracted using a QIAamp Viral RNA Mini Kit (Qiagen Inc.). Thereafter, Accuscript High Fidelity 1st strand cDNA kit (Agilent Technologies, Santa Clara, CA) primed with non-ribosomal hexamers (0.6 mM) [14,26] in the presence of SUPERase-In RNase inhibitor (Ambion, Austin, TX) was used for cDNA synthesis [13]. PCR was thereafter performed with One Taq DNA polymerase (New England Biolabs) again primed with non-ribosomal hexamers. The process resulted in the production of PCR amplicons as evidenced on a 2% agarose gel stained with EtBr, and these were purified (Qiagen QIAquick PCR purification kit), then A-tailed with Taq DNA polymerase (New England Biolabs) as mentioned above. The A-tailed PCR amplicons were subsequently TA-cloned using a pCR2.1 vector with one shot TOP10 chemically competent *E*. *coli* cells, and the inserts (5 to 40 clones from each plasma sample, depending on how many TA clones were attained per plasma sample) were Sanger sequenced.

### Sanger sequencing of OROV vRNA

After determining that virus-specific inserts from one plasma sample were presumably from OROV vRNA, a gene walking approach was used to sequence the virus, and 5/3 RACE was used to obtain the 5’ and 3’ end sequences by Sanger sequencing. Briefly, targeted overlapping (tiled) sequences (< 800 bp amplicons) were amplified using Accuscript High Fidelity reverse transcriptase in the presence of SUPERase-In RNase inhibitor (Ambion, Austin, TX), followed by PCR with Q5 high-fidelity DNA polymerase (New England Biolabs) with denaturation steps performed at 98°C. The primers that were optimized for the gene walking sequencing approach are listed in S2 Table; red letters denote nucleotide differences from the corresponding sequence in the reference strain. To obtain the 5′ and 3′ ends of the three OROV genomes, a 5′ and 3′ system for the Rapid Amplification of cDNA Ends (RACE) was used per the manufacturer’s protocols (Life Technologies, Carlsbad, CA, USA); noteworthy, the 3’ vRNA ends were A-tailed using a Poly(A)-Polymerase (ThermoFisher Scientific, USA) prior to 3’ RACE. The PCR amplicons were purified, sequenced bi-directionally using Sanger Sequencing, and the resulting consensus sequences assembled with the aid of Sequencher DNA sequence analysis software v2.1 (Gene Codes, Ann Arbor, MI, USA).

### Evaluation of a pan-bunyavirus RT-PCR screen for OROV in plasma

Since the unbiased amplification and sequencing approach above suggested that OROV vRNA had been detected, an attempt was made to use the multi-bunyavirus species vRNA detection system of Lambert et al. [27] using OROV group-specific primers that amplify a sequence within the small (S) genomic segment of the OROV genome (Group Specific Fs and Group Specific Rs-a primers, as listed in S2 Table, S segment.

### Phylogenetic analyses

All available complete MELV and OROV S, M, and L coding sequences (CDSs) and the corresponding complete CDSs of mosquito-borne viruses previously grouped as California orthobunyavirus species (including MELV) available in the GenBank database (Nov. 2020 search) were retrieved and aligned using MUSCLE v.3.8.31 [28–30]. The presence of phylogenetic signal was investigated by the likelihood mapping analysis of 10,000 random quartets using TREE-PUZZLE v.5.2 [31]. The Pairwise Homoplasy Index (PHI) test for evidence of recombination was implemented in the SplitsTree v.4.14.6 [32] and the substitution saturation assessed by DAMBE v.7.0.14 [33] Phylogeny-based Maximum Likelihood (ML) method was performed through FastTree v.2.1.10–1 [34] and W-IQ-Tree v.1.6.12 [35] using the Markovian model of site-evolution General Time Reversible (GTR) plus CAT with 20 gamma distribution parameters, a mix of Nearest-Neighbor Interchanges (NNI) and Sub-Tree-Prune-Regraft (SPR) (FastTree) algorithms and hill-climbing and stochastic NNI operations 9W-IQ-Tree). The reliability of the nodes was analyzed by the Shimodaira-Hasegawa (SH-like) and SH-aLTR/aBayes/ultrafast bootstrap support values with 1000 replicates.

## Results

### Preliminary RT-PCR screens for CHIKV, DENV, and ZIKV

The plasma samples and cell cultures of this report tested negative for CHIKV, DENV-1 to -4, and ZIKV vRNAs using the ZCD and virus-specific tests, whereas positive and negative controls generated the expected results. No specific alpha- or flavivirus amplicons corresponding to the vRNAs detected by the nested RT-PCR assay of de Morais Bronzoni et al. [23] were formed, suggesting that the viruses in the cell cultures were either alpha- and flaviviruses not commonly encountered in the Americas, or were a different type of virus.

### Screen for malaria parasites

All samples tested negative for malaria parasites.

### Isolation and identification of MELV

**Fig 1:**
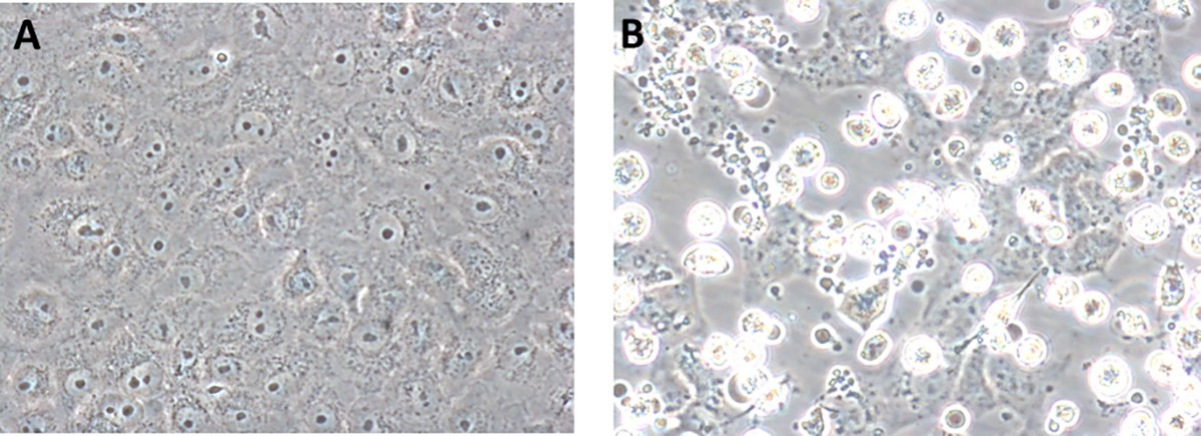
MELV isolation in Vero E6 cells. (A) Mock-infected Vero E6 cells. (B) Vero E6 cells 13 days post-inoculation with plasma from patient A. Apoptotic bodies and dead cells that have detached from the growth surface are evident. Original magnification 400X.

Virus-induced cytopathic effects were observed in LLC-MK2 and Vero E6 cells 10–15 dpi. with plasma from five case patients (all negative by PCR for CHIKV, DENV, and ZIKV). The CPE consisted of the formation of apoptotic bodies in a majority of infected cells and the spindling of some cells, leading to disruption of the cell monolayer (Fig 1), as has been observed for other bunyaviruses [10,36].

**Fig 2:**
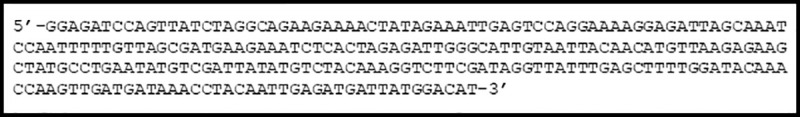
MELV sequence in plasmid insert.

The sequence of the 249 bp insert in one out of 40 plasmids that were obtained after TA-cloning of amplicons from unbiased RT-PCR amplification of the virus genome revealed that the insert had 100% identity with the corresponding sequence of the MELV reference strain TRVL 9375’s L segment (GenBank NC_043634.1) (Fig 2). The inserts in the other 39 plasmids corresponded to African green monkey-derived sequences from the Vero E6 cells used for virus culture.

Full-length coding sequences for the three MELV genome segments were subsequently obtained from viruses isolated from 4 of the 5 patients, using both Sanger and NGS sequencing (the 5th isolate was only partly sequenced for economic reasons; its sequences were similar to the others from Haiti). GenBank accession numbers for the sequences and nucleotide identity among the genome sequences isolates and the reference strain are given in Table 1.

### Demographic and clinical data available for six children with orthobunyavirus infections

Clinical data were available for four of the five with MELV infections children (Table 2). Of the five case patients found to be infected with MELV, the initial case, a four-year-old boy, presented to the Christianville Foundation school clinic in August 2014 with a history of self-reported fever and abdominal pain; he had no complaints of headache, and no visible rash. Three months later, the other four MELV-positive case patients presented to the clinic, three with documented fever above 37.5°C. The average age of the five children with MELV infection was 6.3 years. The five children with MELV infections were from three of the four school campuses that comprise the Christianville Foundation school system; while the three schools are within a 10 km radius of each other, there is no movement of children among campuses. Lymphocytosis was recorded in four out of the five children with MELV infections, with three showing evidence of neutropenia (Table 2). The four children with MELV infections for which clinical data was available complained of abdominal pain, with lymphadenopathy noted on exam in two patients. Headache was reported by one case patient. There was no mention of meningeal signs on exam.

**Table 2:** Demographic and clinical data for six orthobunyavirus infection cases.

Child ID	Clinic visit date (yr. 2014)	Virus	Sex/Age	School	Temp (°C)	Hb^b^ (mg/dL)	WBCc (no./μL)	Neut.^d^ (%)	Signs and Symptoms
1	05 Aug.	MELV	M/4	1	36.2	12.3	7,400	32	Abdominal pain.
2	05 Nov.	MELV	F/5	3	37.9	11.2	10,200	59	Abdominal pain, chills, loss of appetite.
3	19 Nov.	MELV	M/13	1	40.5	12.3	11,000	26	Abdominal pain, headache, lympadenopathy, cough.
4	16 Nov.	MELV	M/6	2	N/A^a^	13.0	7,900	29	N/A
5	25 Nov.	MELV	M/6	1	39.9	11.6	13,000	70	Abdominal pain, headache, lympadenopathy, sores, blisters, impetigo.
6	26 May	OROV	M/6	4	N/A	N/A	N/A	N/A	N/A

^a^N/A, not available.

^b^Hb, hemoglobin.

^c^White blood cell (WBC) count (no. WBC/μL).

^d^Neutrophil count (% of WBC).

### Quantification of MELV in plasma

To gain further insights regarding the state of viremia in the five patients with MELV infections, we quantified the amount of virus in the plasma samples. As shown in Table 3, the infectious MELV titers, expressed as TCID_50_/ml, of MELV were relatively low, ranging from 2.68E+02 to 2.15E+03 TCID_50_/ml. The positive control consisted of MELV propagated in Vero E6 cells.

**Table 3:** MELV titers in plasma.

Sample	TCID_50_/ml
Patient 1 plasma	2.15E+03
Patient 2 plasma	2.15E+03
Patient 3 plasma	3.73E+02
Patient 4 plasma	2.68E+02
Patient 5 plasma	4.64E+02
Positive control	6.31E+04
Negative control	0

### Identification of OROV

**Fig 3:**
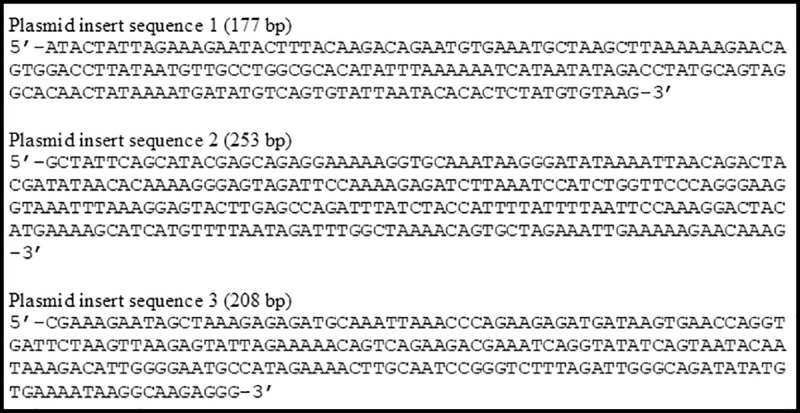
OROV sequences in plasmids.

As noted in the Materials and Methods section, for samples that did not show evidence of growth in LLC-MK2 or Vero E6 cells, we returned to the original case patient plasma sample and did a final screen for vRNA by using an unbiased sequence amplification approach. Upon sequence analyses of the inserts in plasmids resulting from our unbiased sequence amplification approach, three sequences from a sixth case patient (Table 2) showed identity with OROV (Fig 3). Identity was further confirmed by RT-PCR of RNA extracted from the plasma sample with OROV using Group Specific primers, albeit with some modification of the reverse primer S2 Table. A gene-walking approach was then used to obtain the consensus sequences of the virus’ genome segments by Sanger sequencing. The virus associated with the three OROV genomic sequences was designated Oropouche virus/Homo sapiens/Haiti-1/2014, and the GenBank accession nos. and rnt segment lengths are listed in Table 4. The L segment contains a single open reading frame (ORF) encoding a 2,252 aa RNA-dependent RNA polymerase, the M segment contains a single ORF encoding a 1,420 aa polyprotein, and the S segment contains 2 ORFs encoding a 231 aa nucleoprotein and a 91 aa non-structural protein. The complete L genome sequence of OROV Homo sapiens/Haiti-1/2014 has 99.68% nt identity with that of OROV BeH759146 (GB no. KP691630.1). Its complete M genome has 99.89% nt identity with that of OROV BeH759021 (GB no. KP691607.1). Finally, the S genome has 98.33% nt identity with that of OROV TRVL9760 (GB no. KP026181.1).

**Table 4:** GenBank accession nos. and segment lengths (rnt) of OROV Homo sapiens/Haiti-1/2014.

Segment	GenBank Accession No.	Segment length (rnt)
Large (L)	MN264267	6852
Medium (M)	MN264268	4835
Small (S)	MN264269	958

### Demographic data for OROV patient

The one child with OROV presented in May, 2014 (Table 2). He was diagnosed clinically as having CHIKV infection; no further clinical details were available for this case patient.

### MELV and OROV phylogeny

Likelihood mapping analysis showed excellent phylogenetic signal (noise <1% in each genome segment), and a separate phylogenetic analysis of each was carried out in order to maximize the representation of currently available sequences.

**Fig 4:**
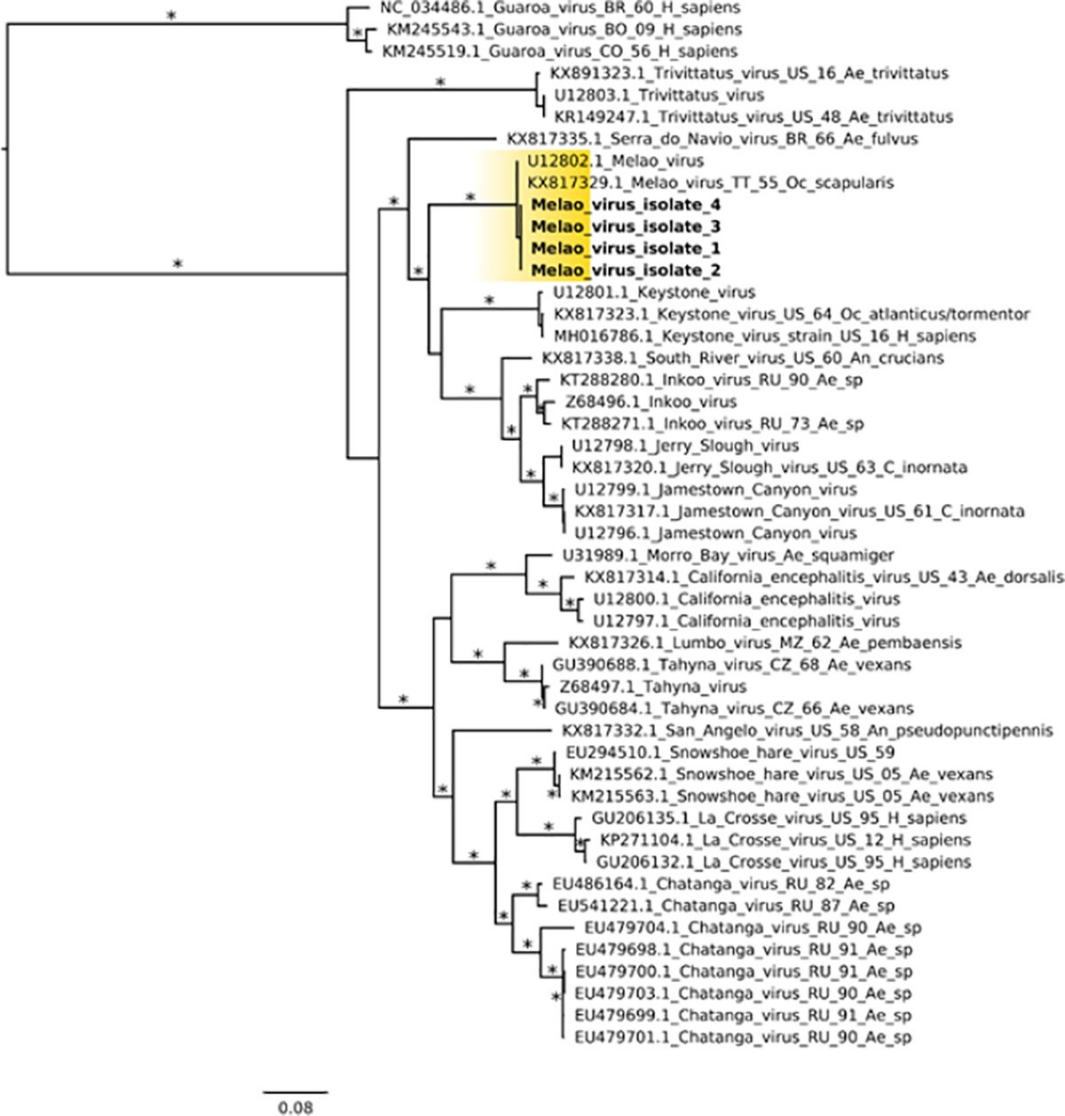
Maximum likelihood (ML) tree based on the Small genome segment of MELV and other California serogroup orthobunyavirus isolates. MELV isolates from this study are highlighted in yellow color; GenBank accession numbers for the corresponding small genome sequences are: 1 (MN264272); 2 (MN268724), 3 (MN268727), and 4 (MN268730). Asterisks represents Shimodaira-Hasegawa (SH-like) support values equal or greater than 75%. Branch lengths are drawn in scale of nucleotide substitutions per site according to the bar in the figure. Abbreviations: BR, Brazil; BO, Bolivia; CO, Colombia; CZ, Czech Republic; MZ, Mozambique; RU, Russia; TT, Trinidad and Tobago; US, United States. Mosquito genera: *Ae*., *Aedes; An*., *Anopheles; C*., *Culiseta; Oc*., *Ochlerotatus*.

**Fig 5:**
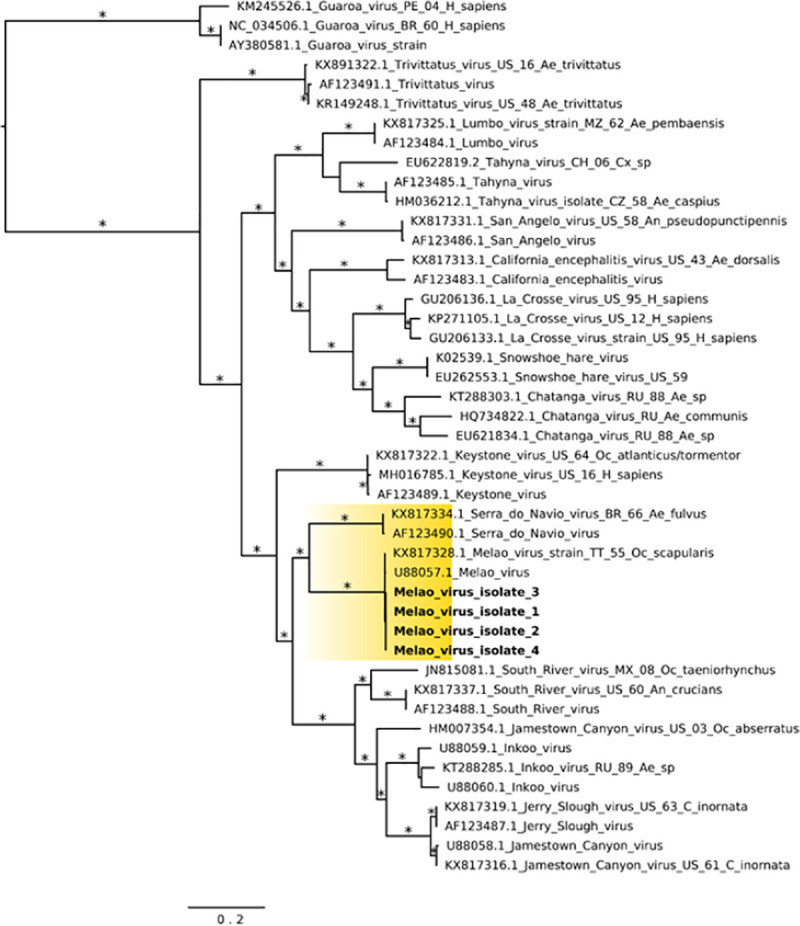
Maximum likelihood tree based on the Medium segment of MELV and other California serogroup orthobunyaviruses isolates. MELV isolates from this study are highlighted in yellow color; GenBank accession numbers for the corresponding small genome sequences are: 1 (MN264271); 2 (MN268723), 3 (MN268726), and 4 (MN268729). Asterisks represents Shimodaira-Hasegawa (SH-like) support values equal or greater than 75%. Branch lengths are drawn in scale of nucleotide substitutions per site according to the bar in the figure. Abbreviations: BR, Brazil; CH, China; CZ, Czech Republic; MX: Mexico; PE, Peru; RU, Russia; TT, Trinidad and Tobago; US, United States. Mosquito genera: *Ae*., *Aedes; An*., *Anopheles; Cx*., *Culex; C*., *Culiseta; Oc*., *Ochlerotatus*.

**Fig 6:**
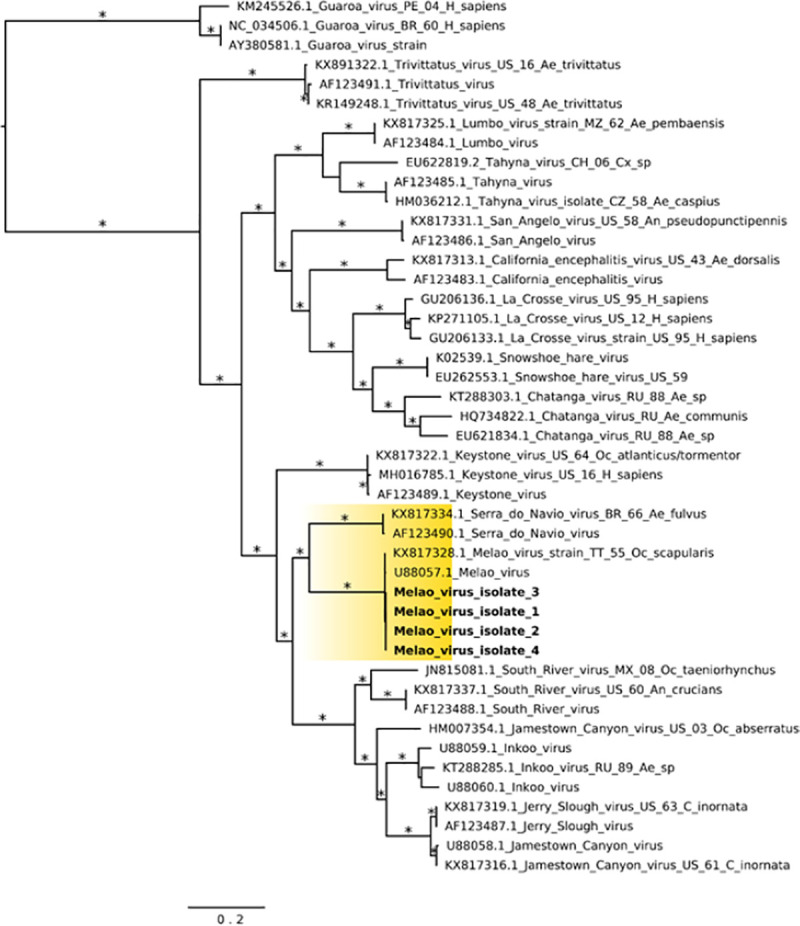
Maximum likelihood tree based on the Large segment of MELV and other California serogroup orthobunyaviruses isolates. MELV isolates from this study are highlighted in yellow color; GenBank accession numbers for the corresponding small genome sequences are: 1 (MN264270); 2 (MN268722), 3 (MN268725), and 4 (MN268728). Branches are labeled with Shimodaira-Hasegawa (SH-like) support values equal or greater than 75%. Branch lengths are drawn in scale of nucleotide substitutions per site according to the bar in the figure. Abbreviations: BR, Brazil; CZ, Czech Republic; FI, Finland; MZ, Mozambique; PE, Peru; RU, Russia; TT, Trinidad and Tobago; US, United States. Mosquito genera: *Ae*., *Aedes*; *An*., *Anopheles*; *C*., *Culiseta*; *L*., *Lepus; Oc*., *Ochlerotatus*.

The Haitian MELV sequences cluster within a single clade together with the previously reported isolate from Trinidad and Tobago. Given the paucity of other available MELV sequence data, comparisons with the Haitian strains were based on an analysis of sequence data from other orthobunyaviruses within the California serogroup. As previously described [6], inferences from Maximum Likelihood (ML) analysis show the segregation of California serogroup viruses into the following groups: (a) Trivittatus virus (TVTV) group, (b) California encephalitis virus (CEV) group, and (c) MELV. Our ML tree analysis of the S-segment infers Serra do Navio virus (SDNV) as the ancestor to the Haitian MELV isolates, which clusters with Keystone virus (KEYV), followed by another distinct clade which includes South River virus (SORV), Inkoo virus (INKV), Jerry Slough virus (JSV) and JCV (Fig 4). Furthermore, data from M- and L-segment phylogenetic trees showed nearly identical topologies (Figs 5 and 6).

**Fig 7:**
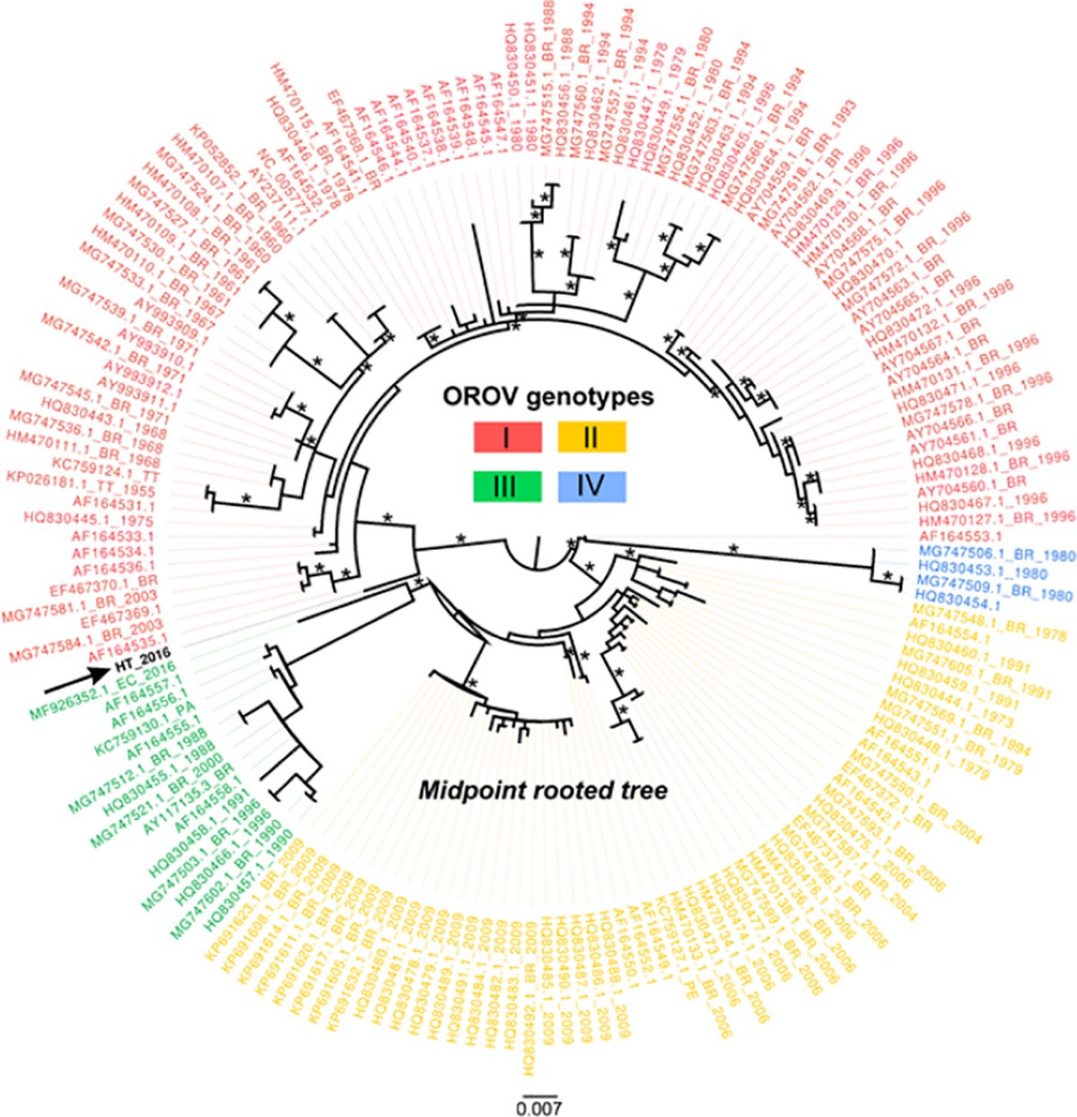
Maximum likelihood (ML—IQ-Tree) midpoint rooted tree based on 166 representative Small segment sequences (693 sites) of different OROV strains. Tips are colored according to genotype. The OROV sequence of this study (GenBank no. MN264269) is identified by the arrow and bolded name. Tip labels refer to GenBank accession code, country of origin (BR, Brazil; PE, Peru; EC, Ecuador; PA, Panama; HT, Haiti; TT, Trinidad and Tobago), and year of isolation. Asterisks indicate SH-aLTR/aBayes/ultrafast bootstrap support values. Only values equal or greater than 75% are shown. Branch lengths are drawn in scale of nucleotide substitutions per site according to the bar in the figure.

**Fig 8:**
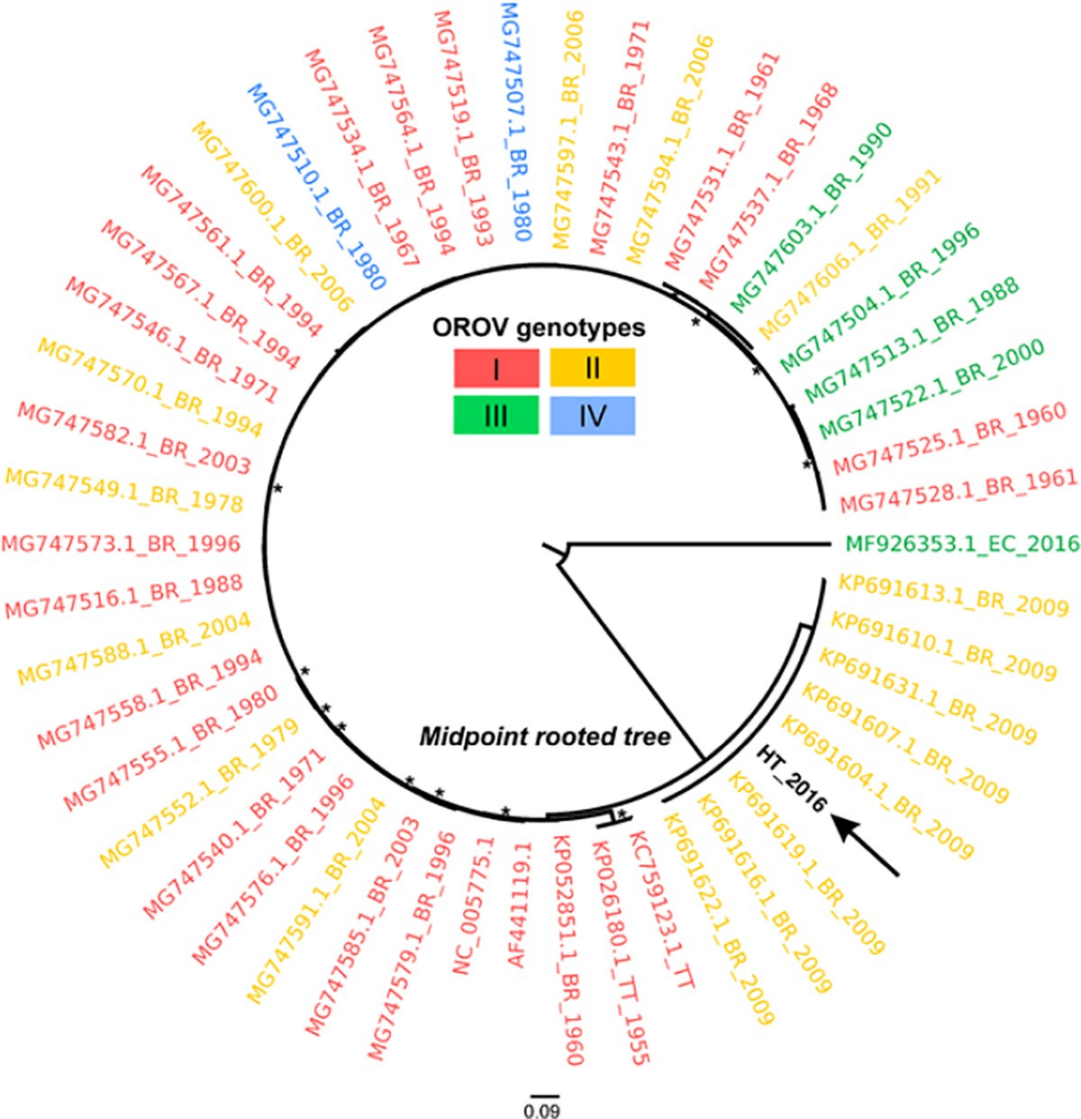
Maximum likelihood (ML) midpoint rooted tree based on 50 representative Medium segment sequences (4357 sites) of different OROV strains. Tips are colored according to genotype. The OROV sequence of this study (GenBank no. MN264268) is represented by the arrow and bolded name. Tip labels represents GenBank accession code, country of origin (BR, Brazil; PE, Peru; EC, Ecuador; HT, Haiti; TT, Trinidad and Tobago), and year of isolation. Asterisks indicate SH-aLTR/aBayes/ultrafast bootstrap support values. Only values equal or greater than 75% are shown. Branch lengths are drawn in scale of nucleotide substitutions per site according to the bar in the figure.

**Fig 9:**
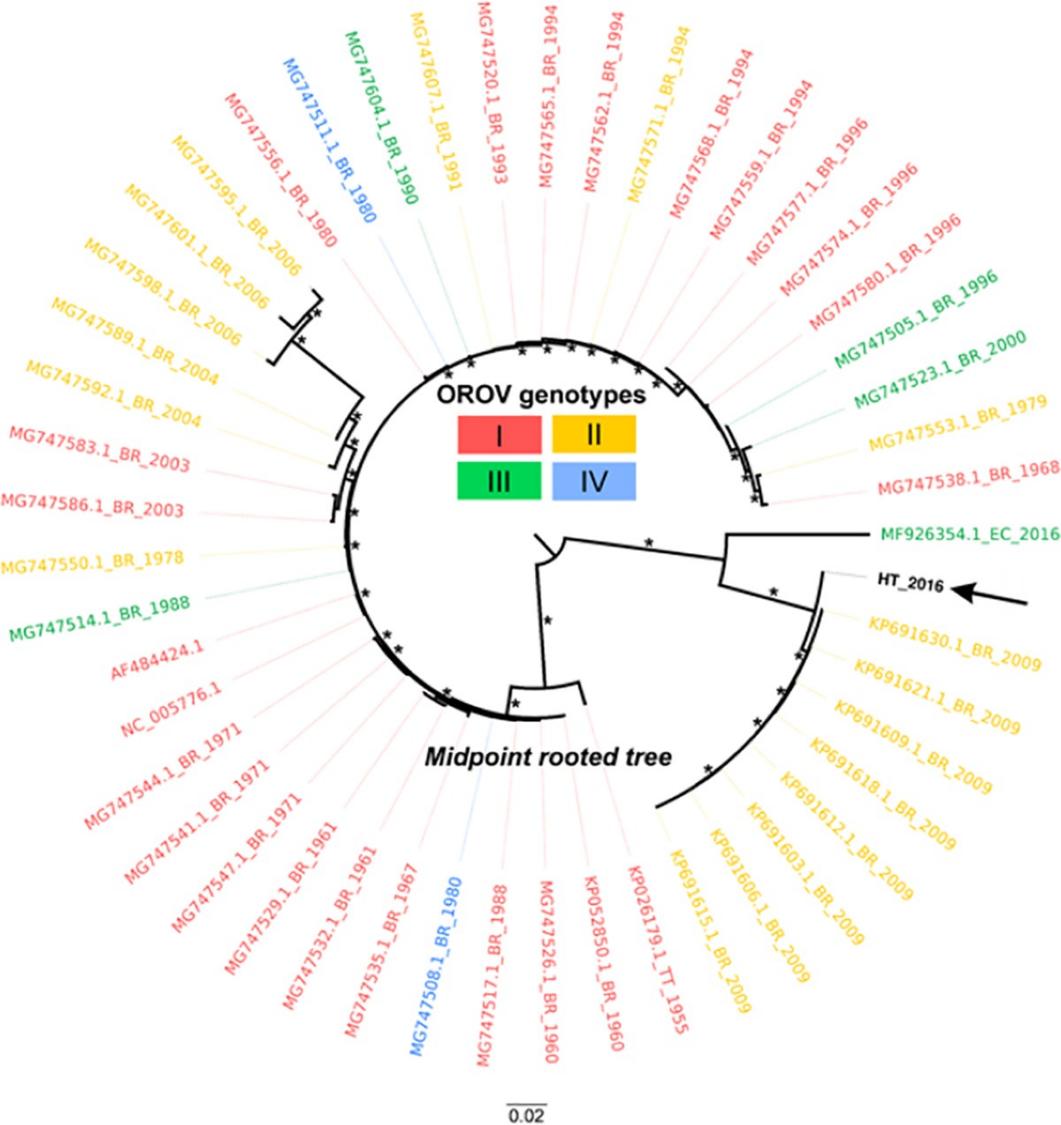
Maximum likelihood (ML) midpoint rooted tree based on 49 representative Large segment sequences (6830 sites) of OROV strains. Tips are colored according to genotype. OROV human isolate from this study (GenBank no. MN264267) is represented by the arrow and bolded name. Tip labels represents GenBank accession code, country of origin (BR, Brazil; PE, Peru; EC, Ecuador; HT, Haiti; TT, Trinidad and Tobago), and year of isolation. Asterisks assumes SH-aLTR/aBayes/ultrafast bootstrap support values. Only values equal or greater than 75% are shown. Branch lengths are drawn in scale of nucleotide substitutions per site according to the bar in the figure.

Sufficient OROV sequence data are available from public databases to permit comparisons with the Haiti sequence data. In the maximum likelihood inferred from the S segment of the OROV genome, commonly used for sub-classification of OROV strains in genotypes [37], the new Haitian isolate appears to be paraphyletic to a genotype I sequence from Brazil (Fig 7). Since only one sequence from Haiti is available, any inference should be taken with caution, but the phylogeny does suggest a possible role of the Caribbean region in the origin and dissemination of this virus that deserves further investigation. Similar analyses of the OROV M and L genome sequences also suggest a relationship to an OROV sequence from Brazil (Figs 8 and 9).

## Discussion

The causes of acute febrile illnesses in Haiti are understudied. Recent waves of concurrent outbreaks of CHIKV, DENV and ZIKV in the country, along with the sporadic reports of infections by neglected tropical viruses [14,21], point out the need for ongoing studies of virus circulation within Haiti and the Caribbean to better understand the causes of undifferentiated acute febrile illnesses, and the potential for future arbovirus epidemics. It is unknown whether MELV, a long-neglected tropical virus, has the potential to cause serious health outcomes in humans. We did observe what appeared to be a consistent clinical syndrome among our case patients, with fever, abdominal pain, and lymphadenopathy. However, these symptoms and signs are relatively non-specific, and do not provide clinicians with the ability to make a diagnosis outside of a research setting. While there have not been well-documented prior reports of human illness due to MELV, there are scattered reports of neutralizing antibodies found in serum collections, including collections from humans and horses in Argentina (where neutralizing antibodies were found in 2 of 20 human samples tested against a MELV “subtype” identified by compliment fixation) [10], and from humans and cattle in Sri Lanka [11]. Given the close relationship of MELV to other viruses traditionally placed within the California serogroup, it may well be that these serologic findings reflect cross-reaction with antibodies to other orthobunyaviruses, which are known to have a global distribution. Nonetheless, based on our findings we think it is reasonable to assume that MELV can cause a febrile syndrome in children; further studies will be needed to define the rates of asymptomatic infection in humans, and determine if it can cause the neuroinvasive syndromes reported in association with other, closely related orthbunyaviruses, such as JCV.

Our identification of MELV in samples from children at three different campuses of the Christianville Foundation school system strongly suggests that the virus was being actively transmitted within the local community in the latter part of 2014. Three mosquito-species have been documented to carry MELV: *Ae*. *serratus*, *Ae*. *scapularis* and *Ps*. *ferox* [36]. Two of these are prevalent in Haiti, *Ae*. *scapularis* and *Ps*. *ferox*, and all three species are prevalent in the United States [38,39]. *Ps*. *ferox* and *Ae*. *serratus* are both woodland mosquitoes that occasionally feed on man, whereas *Ae*. *scapularis* are low-land freshwater mosquitoes that are primarily anthropophilic. It is not yet known which, if any, of those three species are the main vector of MELV in Haiti. Given the lack of diagnostics for MELV, it is also unclear how widespread the infection is. With initial isolation from a mosquito in Trinidad, and now isolation from children in Haiti, it is reasonable to postulate a relatively wide distribution in the Caribbean. Further delineation of its range will require additional sequence-based studies.

In contrast to MELV, which had not previously been regarded as a human pathogen, OROV is recognized as an important arboviral cause of febrile illness, with links to major outbreaks in Brazil and other parts of South America [40]. In a recent report of an outbreak of 46 cases in the Peruvian Amazon [41], major symptoms were headache (87% of patients), myalgias (76%), arthralgias (65%) and retroorbital pain (61%); while not that common, occurrence of meningoencephalitis has also been reported. The combination of fever and arthralgias is suggestive of infection with CHIKV, resulting in misdiagnosis as chikungunya fever in OROV outbreaks, as in our case in Haiti. It is not clear why we were unable to isolate OROV from the plasma of the patient in which the virus’ RNA was detected. The plasma sample had been freeze-thawed many times as different virology and molecular detection analyses were performed. But it may have also been due to residual vRNA present in white blood cells or in virus factories or in virions already inactivated by some component of the immune system. While our data suggests the presence of OROV in Haiti, we acknowledge the following limitations: (1) the children’s travel histories were not documented so we cannot tell if the infection was acquired in Haiti, and (2) no additional cases of OROV were detected within our student cohort other than this single case. Follow-up studies are thus needed to further evaluate the presence of OROV in Haiti. A subtle but important find in our work was that the Group Specific reverse primer of a commonly used test for the detection of OROV vRNA (Group Specific primer Rs, S2 Table, S segment primers) did not work for the OROV vRNA we detected and had to be replaced with Group Specific primer Rs-a (S2 Table, S segment primers). We posit that the presence of OROV vRNA may have been missed in surveillance projects that relied solely on these primers to screen for the virus.

Unlike MELV, OROV is transmitted within Central and Latin America through biting midges, particularly the anthropophilic species *Culicoides paraensis* [12,40]. Though OROV was isolated from *Ae*. *serratus* and *Cx*. *quinquefasciatus*, it is unknown which vector is responsible for its transmission in Haiti. Other species within the culicoid family of flies have been reported in Haiti [42,43] but it is unknown if *C*. *paraensis* is present there. Our phylogenetic study suggests that the OROV strain we identified in Haiti may be a divergent lineage related but distinct from other, previously identified, genotype I sequences from Brazil. This finding deserves further investigation to assess both the role of the Caribbean in the dissemination of this virus and the potential existence of additional unknown genotypes.

A weakness in this particular study is that serology follow-up was not possible. Apart from lack of IRB approval for a serology study, the UF laboratory at the study site in Gressier, Haiti, is no longer in operation. We thus do not have access to the study population for follow-up serology and related studies. It would have been interesting and informative to assess the immune status of the patients of this work to MELV and OROV, and that of their peers and others living in the area, and to gain insights whether MELV and OROV continue to circulate there.

Orthobunyaviruses are distributed globally. Specific viruses within the genus are known to have the potential for causing serious human illness, including meningoencephalitis-neuroinvasive disease [4,44]. However, we still know very little about the human pathogenic potential for the majority of orthbunyavirus strains, due in large part to the number and diversity of species and the lack of readily accessible diagnostic tools for their identification. The cases which we report were identified only because infected children were part of an intensive study of viral causes of febrile illness in Haiti, which included use of virus cultures and unbiased sequencing techniques. Our findings document the occurrence and spread of these orthobunyaviruses within the Caribbean region and highlight the critical importance of surveillance (with unbiased sequencing approaches) to identify outbreaks/epidemics caused by these and other emerging viruses.

## Supporting information

S1 Table Primers for the Sanger sequencing of Melao virus/Homo sapiens/Haiti/2014 isolates 1–4. (DOCX)

S2 Table Primers for Sanger sequencing and for the detection of Oropouche virus/Homo sapiens/Haiti-/2014. (DOCX)
