# Supplementary material for: Orthobunyaviruses in the Caribbean: Melao and Oropouche virus infections in school children in Haiti in 2014
Source: PLoS Negl Trop Dis. 2021 Jun 16;15(6):e0009494. doi: 10.1371/journal.pntd.0009494 (PMC8238191; doi:10.1371/journal.pntd.0009494)
Supplement: S1 Table — (DOCX) [file pntd.0009494.s001.docx]

**S1 Table**

**Primers for the Sanger sequencing of Melao virus/Homo sapiens/Haiti/2014 isolates 1 – 4.**

| **Segment** | **Primer** | **Sequence (5’-3’)** | **Nt position in NC_043634.1** |
| --- | --- | --- | --- |
| L | 5’ RACE roligo | rArGrC rArUrC rGrArG rUrCrG rGrCrC rUrUrG- rUrUrG rGrCrC rUrArC rUrGrG | N/A |
|  | 5’ RACE - F | TCGTAGCTCAGCCGGAACAACCGGATGACC | N/A |
|  | 5’ RACE - R | GGTCTGATATCAAGTATTATATC | 248 - 226 |
|  | 1Fl | CATGACTATTTTGGCAAAGAACTATGC | 157 - 183 |
|  | 1Rl | CCCTCTGAACCATTAAGTCCCACCCCTC | 919 - 892 |
|  | 2Fl | GCCACAGGCGATTTTAAGCAAC | 850 - 871 |
|  | 2Rl | CAATGTTGAGAAGTCTGAAATACATTGCC | 1599 - 1571 |
|  | 3Fl | GGATACATTTGACATTATGGCAG | 1527 - 1549 |
|  | 3Rl | GTGTCACACTACCAGGAAACCATATG | 2269 - 2244 |
|  | 4Fl | GACTATGACATAACTCAAAAAGGTATAAAGG | 2194 - 2224 |
|  | 4Rl | CTCCTACAAATATCTCCCTATC | 2947 - 2926 |
|  | 5Fl | GGACATGATGGTGAACCATAAG | 2859 - 2880 |
|  | 5Rl | GTATTGAGCCTTCCAAGAATG | 3592 - 3572 |
|  | 6Fl | GCAGTTATGTCCATAGTTGTGCAATG | 3512 - 3537 |
|  | 6Rl | GGATCCATCTCGGCATCTAATACAAG | 4289 - 4264 |
|  | 7Fl | GGGATGTGAATGTATTAGATG | 4208 - 4228 |
|  | 7Rl | GGATCTGCGCCCTGCACATCTG | 4982 - 4961 |
|  | 8Fl | CACCATTCTCCTGCACTCGTTTTGC | 4915 - 4939 |
|  | 8Rl | CCTCACCAATTATAGTTATAGATC | 5689 - 5666 |
|  | 9Fl | CATCTAGACATTTAGATATGGGAGC | 5612 - 5636 |
|  | 9Rl | CTGTAAGTCATATGTTTCTCTCCCTTC | 6389 - 6363 |
|  | 10Fl | CACAGAAGGGGAAGCAATACATGC | 6309 - 6332 |
|  | 10Rl | CTGAATTATGTTCTTTGAAATCTTCAAAC | 6877 - 6849 |
|  | 3’ RACE - F | GGAGACGCAAAGAGATTTCAG | 6771 - 6791 |
|  | T25 | TTTTTTTTTTTTTTTTTTTTTTTTT | N/A |
|  | | | |
| **Segment** | **Primer** | **Sequence (5’-3’)** | **Nt position in NC_043633.1** |
| M | 5’ RACE roligo | rArGrC rArUrC rGrArG rUrCrG rGrCrC rUrUrG- rUrUrG rGrCrC rUrArC rUrGrG | N/A |
|  | 5’ RACE - F | TCGTAGCTCAGCCGGAACAACCGGATGACC | N/A |
|  | 5’ RACE -1Rm | GGCAGACTTCTGTAACTGCG | 178 - 159 |
|  | 1Fm | CCAGTATACCAGA**A**ATGCTTCC | 100 - 121 |
|  | 1Rm | CCACATAACTTACATTTCTTGC | 821 - 800 |
|  | 2Fm | CAGGTTCTATAGCAAACTCTATATGC | 626 - 651 |
|  | 2Rm | CATCTTCATACTGCCCACAAGTGC | 1345 - 1322 |
|  | 3Fm | GTGAATGTGACATGTACCATG | 1250 - 1270 |
|  | 3Rm | GTCCCTCTGAAAGCTTTGTGAAAGG | 2015 - 1991 |
|  | 4Fm | GGATGTTACTTGAATAAACAAG | 1933 - 1954 |
|  | 4Rm | CTTTATCATAATCTCTATGCACTTC | 2704 - 2680 |
|  | 5Fm | CACAGGAACCTGCGATGTATTAGGC | 2607 - 2631 |
|  | 5Rm | GTCAGTGGTTTTCCTATAAACG | 3363 - 3342 |
|  | 6Fm | GATGGCTGCGTATTTGGGTCATG | 3292 - 3314 |
|  | 6Rm | CTGCCTCGACTTTCGTGTTAC | 4081 - 4061 |
|  | 7Fm | GCCATGACAAACCTGCTTCTTCG | 4022- 4044 |
|  | 7Rm | CTTGCTTACCTAATCTTCATTTCC | 4391 - 4368 |
|  | 3’RACE Fm | GACACTCTAAAGCAACATGAAG | 4333 - 4354 |
|  | T25 | TTTTTTTTTTTTTTTTTTTTTTTTT | N/A |
|  | | | |
| **Segment** | **Primer** | **Sequence (5’ – 3’)** | **Nt position in NC_043635.1** |
| S | 5’ RACE roligo | rArGrC rArUrC rGrArG rUrCrG rGrCrC rUrUrG- rUrUrG rGrCrC rUrArC rUrGrG | N/A |
|  | 5’ RACE - F | TCGTAGCTCAGCCGGAACAACCGGATGACC | N/A |
|  | 5’RACE-1Rs | CATGCGCAATTGTAAATGCCAGATAC | 162 - 137 |
|  | 1Fs | GTGCAAATGGATTTGATCCAG | 109 - 129 |
|  | 1Rs | CCTTACCCCACTTCAGCTGCTCTAC | 720 - 696 |
|  | 2Fs | GTAGAGCAGCTGAAGTGGGGTAAGG | 608 - 630 |
|  | 2Rs | GTATTCAGTAGTGTGCTCCACTG | 969 - 953 |
|  | 3’ RACE Fs | GTGGGTGGTAGGGGACAGCAAAG | 893 - 915 |
|  | T25 | TTTTTTTTTTTTTTTTTTTTTTTTT | N/A |
