# Supplementary material for: Orthobunyaviruses in the Caribbean: Melao and Oropouche virus infections in school children in Haiti in 2014
Source: PLoS Negl Trop Dis. 2021 Jun 16;15(6):e0009494. doi: 10.1371/journal.pntd.0009494 (PMC8238191; doi:10.1371/journal.pntd.0009494)
Supplement: S2 Table — (DOCX) [file pntd.0009494.s002.docx]

**S2 Table**

**Primers for Sanger sequencing and for the detection of Oropouche virus/Homo sapiens/Haiti-/2014.**

| **Segment** | **Primer** | **Sequence (5’-3’)** | **Nt position in KP026179.1** |
| --- | --- | --- | --- |
| L | 5’ RACE roligo | rArGrC rArUrC rGrArG rUrCrG rGrCrC rUrUrG- rUrUrG rGrCrC rUrArC rUrGrG | N/A |
|  | 5’ RACE - F | TCGTAGCTCAGCCGGAACAACCGGATGACC | N/A |
|  | 5’ RACE - R | GCAGCTCTGCAAAATTCTCGAGAAAAG | 177 - 151 |
|  | 1Fl | AGTAGTGTACTCCTATTTCAAAAC | 1 - 24 |
|  | 1Rl | CTGTTCTGC**T**TCTGGCATTGAATTC | 694 - 670 |
|  | 2Fl | GAGAATTCACAATGACATTGCCATGGATTG | 591 - 620 |
|  | 2Rl | GGCTTATCAGTATTTATGTCA | 1350 - 1330 |
|  | 3Fl | GAAGA**T**AGAATACA**C**TTAATGAAAG | 1,256 – 1,280 |
|  | 3Rl | CATGATCATATA**G**CG**C**GAAGGCTC | 2,002 - 1,979 |
|  | 4Fl | CTAGGATTTTCATTCTTTACATC | 1,922 – 1,944 |
|  | 4Rl | GTGATTTCATCTAAGAATGCTG | 2,679 – 2,658 |
|  | 5Fl | GAGAAAAAC**C**AAAAATGAT**G**CAAAGA | 2,593 – 2,618 |
|  | 5Rl | GTTTGTCATTAA**C**CCAATTAT**A**TCATTTG | 3,394 - 3,366 |
|  | 6Fl | GCA**A**AAAAGGCTTATAATGCCTGATG | 3,301 – 3,326 |
|  | 6Rl | GTGACATTTCCTGCTTCTAAACCAG | 4,068 – 4,044 |
|  | 7Fl | CCATTCTTCCCTACTAACAATAGAA | 3,959 – 3,983 |
|  | 7Rl | GTCTTGACATC**A**TTTATATCCAATG | 4,719 – 4,695 |
|  | 8Fl | GAAGAACTAGATACAAT**C**ATAGGTCG | 4,616 – 4,641 |
|  | 8Rl | CAGCAAAATGTGCTACTAGTCT**C**AGTGC | 5,330 – 5,303 |
|  | 9Fl | GGTACATAACACATTACTTAAAAAATATAGTG | 5,208 – 5,239 |
|  | 9Rl | GATTCTTAACTTTGGAAAGTATAGG | 5,975 – 5,961 |
|  | 10Fl | TATCAAAACT**T**TA**T**CAACAAGGACG**A**CCTATAC | 5,939 – 5,957 |
|  | 10Rl | GTTTGGAAATCTTCAATTAATAATATGAACTC | 6,654 – 6,623 |
|  | 11Fl | GGACAGAGGT**T**ATTGA**G**TTCATATTATTAATTGAAG | 6,609 – 6,644 |
|  | 11Rl | AGTAGTGTGCTCCTATTTAGAAACAAACAC | 6,852 – 6,823 |
|  | 3’ RACE - F | GTATGAAGTCACA**T**TTCTGTTCAAAAGCACACAG | 6,675 – 6,708 |
|  | T25 | TTTTTTTTTTTTTTTTTTTTTTTTT | N/A |
|  | | | |
| **Segment** | **Primer** | **Sequence (5’-3’)** | **Nt position in KP026180.1** |
| M | 5’ RACE roligo | rArGrC rArUrC rGrArG rUrCrG rGrCrC rUrUrG- rUrUrG rGrCrC rUrArC rUrGrG | N/A |
|  | 5’ RACE - F | TCGTAGCTCAGCCGGAACAACCGGATGACC | N/A |
|  | 5’ RACE -1Rm | GAGGTTACCACCAGCAAAACAGCGG | 133 - 109 |
|  | 1Fm | AGTAGTGTACTACCAGCAACAAACAG | 1 - 26 |
|  | 1Rm | GGATAAAATAACGGCAGGAGCAGGTATG | 759 - 732 |
|  | 2Fm | GCTAATAATTCTGACATTATATATCTTTGC | 661 – 690 |
|  | 2Rm | GCACAGATTACAAGTGCAAATAATAAAG | 1452 - 1425 |
|  | 3Fm | CCTAAAGTGGTTTACTCTTGGAC | 1392 - 1414 |
|  | 3Rm | GAGCGGGTGGATCTACCAAGG | 2154 - 2134 |
|  | 4Fm | GATGATTGGAATATTGAAATTTG | 2089 - 2111 |
|  | 4Rm | CTATCTCAGATTCTATTGCTTTTC | 2795 - 2772 |
|  | 5Fm | GACCCACAAAGAATTTAGCACTATG | 2713 - 2737 |
|  | 5Rm | CCAATATCATTGATTTGACCTGTGTA | 3462 - 3437 |
|  | 6Fm | GATAAATTAAGTGCATCCTTTCAG | 3359 - 3382 |
|  | 6Rm | CAATATCTCCCTAAGCCTGAAAATAATC | 4155 - 4128 |
|  | 7Fm | GCAGTTAGATGAATCAAATTACATAAG | 4030 - 4056 |
|  | 7Rm | AGTAGTGTGCTACCAACAACAATTTTTGAC | 4385 - 4386 |
|  | 3’RACE Fm | CAGACATGAAATAGAATATGCCATGGAGC | 4252 - 4280 |
|  | T25 | TTTTTTTTTTTTTTTTTTTTTTTTT | N/A |
|  | | | |
| **Segment** | **Primer** | **Sequence (5’ – 3’)** | **Nt position in KP026181.1** |
| S | 5’ RACE roligo | rArGrC rArUrC rGrArG rUrCrG rGrCrC rUrUrG- rUrUrG rGrCrC rUrArC rUrGrG | N/A |
|  | 5’ RACE - F | TCGTAGCTCAGCCGGAACAACCGGATGACC | N/A |
|  | 5’RACE-1Rs | GTCCGTATCTAGCTTCAAATG | 138 - 113 |
|  | Group Specific Fs | GGCCCATGGTTGACCTTACTTT | 217 - 238 |
|  | Group Specific Rs | ACCAAAGGGAAGAAAGTGAAT | 520 - 500 |
|  | Group Specific Rs-a | ACCAAAGG**A**AAGAAAGTGAA**C** | 520 - 500 |
|  | 1Fs | AGTAGTGTACTCCACAATTC | 1 - 20 |
|  | 1Rs | ctatttcttcacgcatccattgctcAgc | 633 - 606 |
|  | 2Fs | GTGGGGTCCAATTTGCAATGG | 241 - 261 |
|  | 2Rs | AGTAGTGTGCTCCCAATTCAAAAATACG | 958 - 931 |
|  | 3’ RACE Fs | GTGCGAAAGCACAAAAAAAGAACTG | 849 - 873 |
|  | T25 | TTTTTTTTTTTTTTTTTTTTTTTTT | N/A |
